# The Occurrence of Gluten-Related Antibodies, Sensitization to Selected Food Allergens, and Antibodies against Intrinsic Factor in Adult Patients with Diarrhea-Predominant Irritable Bowel Syndrome

**DOI:** 10.3390/jpm13071165

**Published:** 2023-07-20

**Authors:** Joanna B. Bierła, Bożena Cukrowska, Barbara Skrzydło-Radomańska, Beata Prozorow-Król, Anetta Kurzeja-Mirosław, Halina Cichoż-Lach, Katarzyna Laskowska, Agnieszka Sowińska, Emilia Majsiak

**Affiliations:** 1 Department of Pathomorphology, The Children’s Memorial Health Institute, Aleja Dzieci Polskich 20, 04-730 Warsaw, Poland; b.cukrowska@ipczd.pl (B.C.); a.sowinska@ipczd.pl (A.S.); 2 Department of Gastroenterology, Medical University of Lublin, Jaczewskiego 8, 20-950 Lublin, Poland; barbara.radomanska@gmail.com (B.S.-R.); prozorow1@wp.pl (B.P.-K.); anettakm@op.pl (A.K.-M.); lach.halina@wp.pl (H.C.-L.); katarzynalaskowska@go2.pl (K.L.); 3 Department of Health Promotion, Faculty Health of Sciences, Medical University of Lublin, Staszica 4/6, 20-081 Lublin, Poland; emiliamajsiak@umlub.pl

**Keywords:** irritable bowel syndrome, celiac disease, non-celiac gluten sensitivity, allergy, adults, serology

## Abstract

Irritable bowel syndrome (IBS) is a common functional gastrointestinal disorder. Due to the possible overlap of IBS clinical symptoms with gluten-related diseases, food allergies, and autoimmune gastritis (AIG), the aim of this study was to present the frequency of anti-tissue transglutaminase 2 (TTG2) autoantibodies, anti-deamidated gluten peptide (DGP) antibodies, specific immunoglobulin E antibodies (sIgE) to selected food allergens, and anti-intrinsic factor (IF) autoantibodies in adult patients with diarrhea-predominant IBS (IBS-D). The study involved 244 patients (170 women) aged 18–75 years. The antibodies were measured with the use of multiparametric immunoassays. Elevated antibody concentrations, irrespective of the class of tested antibody, occurred in 44 patients (17.6%), including 11 patients (4.5%) with positive DGP antibodies, four patients (1.6%) with TTG2 autoantibodies, six patients (2.5%) with IF autoantibodies, and 31 patients (12.7%) with sIgE to food allergens. Sensitization to gluten, proteins from cow’s milk, and bovine serum albumin was found in 2.1%, 5.3%, and 9.0% of patients, respectively. Our study showed a high percentage of positive results for the tested antibodies in the IBD-D patients, which indicates the need to perform serological tests for CD, food allergies, and AIG in this group of patients.

## 1. Introduction

Irritable bowel syndrome (IBS) is one of the most common and debilitating functional gastrointestinal disorders, with about 11% of prevalence estimated in the global population [1,2,3]. The prevalence of IBS in women is about twice as high as in men, with a worse quality of life and greater severity of pain, abdominal distension, fatigue, and somatization [1]. The pathophysiology of IBS is still unknown, but in the literature, the potential importance of genetic predisposition, altered intestinal motility, intestinal hypersensitivity, psychological disorders, enteric infections, food intolerance, altered intestinal immunity, or changes in gut microbiota are emphasized [4]. The clinical characterization of IBS includes symptoms such as abdominal pain, bloating, and changes in bowel habits (alternating diarrhea and constipation) [1,4]. Since IBS cannot be confirmed by specific laboratory or functional tests, the Rome criteria (recently reintroduced as the Rome IV criteria) are the main tool for making definitive diagnoses [5,6]. According to the Rome IV diagnostic criteria, a patient may be classified as suffering from IBS if they have felt recurrent abdominal pain on average at least 1 day/week in the last 3 months, associated with two or more of the following situations: defecation, a change in the frequency of stool, or a change in the form (appearance) of stool [6]. Based on stool frequency and consistency, IBS has been divided into four main subtypes: diarrhea-predominant (IBS-D), constipation-predominant (IBS-C), mixed bowel habits (IBS-M), and unclassified (IBS-U) [1].

Due to the overlap of IBS symptoms with gluten-related diseases, such as celiac disease (CD), non-celiac gluten sensitivity (NCGS), and gluten allergies, it seems very important to rule out these diseases in IBS patients [7,8,9]. The clinical picture of these patients groups may include persistent gastrointestinal symptoms, like abdominal pain, flatulence, and diarrhea [7,8,9,10]. Moreover, patients can struggle with extra-intestinal manifestations, leading to a reduced quality of life, absenteeism from work, and an increase in healthcare utilization [11,12,13]. These symptoms can include recurrent headaches, sexual dysfunction, recurrent fetal loss, low-birth-weight offspring, aphthous stomatitis, dermatological manifestations, and osteoporosis [14,15,16]. It is also known that diseases such as IBS or CD can manifest themselves as emotional symptoms or comorbid psychiatric disorders, such as chronic fatigue, depression, and polyneuropathy [15,16]. Additionally, psycho-neurological symptoms, like anxiety and depression, can magnify gastrointestinal-symptom perception and make it more salient [17]. Therefore, it is extremely important to perform appropriate differential diagnostics before introducing any treatments, especially dietary interventions.

Gluten is the general name for the water-insoluble prolamin proteins of cereals, which include gliadin in wheat, secalin in rye, hordein in barley, and avenin in oats [18]. Gluten is responsible for the activation of autoimmune processes and the development of CD [19]. The pathogenesis of CD is associated with gluten peptides, arising as products of gluten degradation by gastrointestinal-tract enzymes [20]. Peptides are transferred through the epithelial barrier into the mucosal lamina propria; subsequently, the intestinal enzyme-tissue transglutaminase 2 (TTG2) converts the glutamine residues present in gluten peptides into glutamic acid, and this conversion generates deamidated gluten peptides (DGP) [20]. In genetically predisposed individuals who have genes encoding HLA-DQ2/-DQ8 molecules, DGP strongly binds to these molecules on antigen-presenting cells, activating specific T cells, which, in turn, induce B cells through the production of antibodies directed against TTG2 (TTG2 antibodies) and DGP (DGP antibodies) [21]. The presence of TTG2 antibodies in the immunoglobulin (Ig) A class is a specific marker of autoimmune processes in CD and, currently, these antibodies are referred to as CD specific autoantibodies with high sensitivity and specificity [22,23]. In contrast to TTG2 autoantibodies, which are highly CD-specific, the presence of DGP antibodies generally indicates contact with gluten, so it can be a very good marker for following a gluten-free diet (GFD) in CD patients [24]. Thus, the serological assessment of TTG-2 antibodies and total IgA is currently recommended in children and adolescents as the first step in CD diagnosis [22], and in patients with high concentrations of these antibodies (>10x the upper limit of normal), small-intestine biopsies and histological examinations of intestinal specimens may be waived. It has been shown that the addition of DGP antibodies to the total IgA-plus-TTG2 IgA panel for serological screening does not increase the detection of CD [22]. In adults, the serological testing of antibodies is advised; however, the diagnosis is still based on the histopathological evaluation of biopsies [25,26]. However, in symptomatic adults who are unwilling or unable to undergo endoscopy, the same serological criteria as for children and adolescents can be used [26]. It is worth noting that the histological changes present in CD, such as an increase in the number of intraepithelial lymphocytes, crypt hyperplasia, and atrophy of the intestinal villi, are not features reserved for CD. Similar changes are described in allergies to cow’s milk and gluten [27,28], *Giardia lamblia*, tuberculosis or human-immunodeficiency-virus infections, autoimmune enteropathy, or chronic inflammatory diseases, such as Crohn’s disease, and the changes may be reversible under the influence of immunomodulators, such as thiopurines, combined with biological therapy [29,30].

Gluten also is an allergen that can also activate a cascade of changes leading to the formation of specific antibodies in the IgE class (sIgE), which bind to receptors on the surfaces of mast cells and basophils; this stage is called sensitization [31]. Subsequent repeated contact with a specific antigen leads to the degranulation of effector cells and the release of pro-allergic cytokines and other substances, which are responsible for IgE-dependent food-allergy symptoms [27,31].

In contrast to gluten sensitization/allergy and CD, the pathogenesis of NCGS is unknown. According to the recommendations from Salerno 2015 [32], in order to diagnose NCGS, other gluten-related diseases (allergies and CD) must first be ruled out. There are no specific NCGS markers, but it is known that both antibodies against native gliadin and DPG antibodies may appear in NCGS patients [33].

The aim of the current study was to assess the frequency of CD-specific TTG2 autoantibodies, DPG antibodies, and sensitization to selected food allergens, including gluten, proteins from cow’s milk, and bovine serum albumin (BSA), in a post hoc analysis of adult IBS-D patients included in previous interventional trials. In addition, we assessed the occurrence of antibodies against the intrinsic factor (IF antibodies), which can be associated with autoimmune gastritis (AIG) [34] and was tested in the multiparametric immunoassay used in this study.

## 2. Materials and Methods

### 2.1. Patients and Study Design

This study is a post hoc analysis of the presence of selected antibodies in adult IBS-D patients recruited for three clinical randomized placebo-controlled trials concerning the efficacy of different probiotic preparations. The results two of these trials have been already published [35,36]; the results of the third trial were recently submitted for publication. The patients were recruited between August 2018 and August 2022 at the Department of Gastroenterology, Medical University of Lublin (Poland). Patients aged 18–75 years, diagnosed with IBS-D according to the Rome III criteria [35,36] and Rome IV criteria (the last group of patients) were involved in the study.

During screening visits, the patients underwent physical examination to establish the presence of clinical inclusion and exclusion criteria, which are described in detail in our earlier papers [35,36]. A normal diet was one of the inclusion criteria for the study, and none of the patients were on a GFD or a diet eliminating fermentable oligo-, di-, and monosaccharides and polyols (FODMAP). The exclusion criteria were coexisting severe diseases, such as malignancies, uncontrolled hypertension, uncontrolled diabetes mellitus, serious neurological disorders, psychosis, respiratory disorders (asthma and chronic obstructive pulmonary disease), hyper- or hypothyroidism, hepatic, renal, or cardiac dysfunction, chronic bowel disorders other than IBS, such as inflammatory bowel diseases, CD, gastroenteritis, gastric and duodenal ulcers, and associated constipation, parasitic or bacterial intestinal infestation/infections, diagnosed lactose intolerance, and pregnancy or lactation. 

The IBS severity was assessed with the use of the IBS-SSS scale. The IBS-SSS is a 5-question survey about the severity of abdominal pain (IBS- SSS1), the number of days with abdominal pain over the last 10 days (IBS-SSS2), the severity of abdominal distension (IBS-SSS3), dissatisfaction with bowel habit (IBS-SSS4), and interference with the quality of life over the past 10 days (IBS-SSS5) [37]. Severe IBS was when IBS-SSS score was >300, and moderate was when IBS-SSS score was >175 and ≤300.

After screening for CD, patients with positive results for TTG2 antibodies were referred for endoscopic examination in order to collect intestinal biopsies [26]. Tissue samples were evaluated histopathologically according to the Marsh–Oberhuber scale [38].

Ethical Approval and Trial Registration

All trials were registered at Clinicaltrials.gov, and received the following numbers: NCT04206410, NCT04662957, and NCT05064930. The studies were approved by the local Bioethical Committee of the Children’s Memorial Health Institute (decision number 6/KBE/2018) and Regional Medical Chamber in Lublin (173/2021/KB/VIII) along with the ethical principles set out in the Declaration of Helsinki Guideline on Good Clinical Practice.

### 2.2. Material

For the study, 2.7 mL of blood per patient was collected in vacutainer tubes (powdered glass clot activator), and blood was centrifuged after clot formation. The obtained sera were immediately frozen and stored at −40 °C until laboratory examination.

### 2.3. Methods

Determination of TTG2, DGP, and IF Antibodies

Antibodies in IgA class (Polycheck^®^ Celiac IgA plus total IgA, Biocheck, GmbH, Münster, Germany) and IgG class (Polycheck^®^ Celiac IgG, Biocheck, GmbH, Münster, Germany) were assed with the use of multiparametric immunoassays. Polycheck^®^ Celiac IgA plus total-IgA test allowed detection of concentration of total IgA and antibodies against human recombinant TTG2 (TTG2 IgA) and DGP (DGP IgA) [23]. Using Polycheck^®^ Celiac IgG, TTG2 IgG, DGP IgG, and IF IgG antibodies were detected. The concentrations of antibodies were measured according to manufacturer protocols, described in detail in our previous studies [23,39]. After gentle defrosting, each serum sample was diluted 1:100 and, next, samples examined individually (but simultaneously) for specific set of antibodies. During incubation of the patient sera, antigen–specific IgA or IgG were banded to the corresponding antigens. Non-bounded serum components were removed by washing with phosphate buffer (PBS) with 1% Tween (Sigma, St Louis, MO, USA). Next, alkaline phosphatase conjugated detection antibodies, identifying antigen-bound IgA or IgG were added, and reaction was visualized with 5′bromo-4′chloro-3′indolylphosphate/4′ nitro-bluetetrazolium (BCP/NBT). The color intensity of the lines was proportional to the respective antigen-specific IgA or IgG concentration. Finally, air-dried membranes were scanned and evaluated with the help of Biocheck Imaging Software 5.01.38 Human (Polycheck^®^, Biocheck, GmbH, Münster, Germany). Interpretation was based on each single-antigen identification and quantified according to the calibration curve in each cassette. The antibody concentrations in classes IgA and IgG >0.8 and <0.3 kU/L were considered as positive and negative, respectively. Values ranging from <0.8 to >0.3 kU/L were regarded as equivocal (borderline—very low antibody titer). Total IgA concentrations lower than 0.5 kU/L were considered indicative of IgA immunodeficiency.

Determination of sIgE Antibodies

For the quantitative measurement of sIgE in patient sera, a multiparametric immunoassay (Polycheck^®^, Milk plus Gluten 6, Biocheck, GmbH, Münster, Germany) was used. This assay allowed identification of sIgE antibodies to extract of cow’s milk and components (alpha-lactalbumin, beta-lactoglobulin, casein), gluten, BSA. During incubation of 200 µL of patient sera (1 h on a laboratory shaker at room temperature) allergen-specific IgE banded to corresponding allergens on test membrane. After washing PBS with 1% Tween (Sigma, St Louis, MO, USA) and adding monoclonal ligand-labelled anti-IgE, formed immune complexes were detected by enzyme-labelled anti-ligands and, finally, the reaction was visualized with BCP/NBT substrate. The concentration of specific IgE was measured with the use of Biocheck Imaging Software 5.01.38 Human (Polycheck^®^, Biocheck, GmbH, Münster, Germany). According to the manufacturer protocol, the results higher than 0.35 kU/L were considered positive.

Statistical Analyses

All analyses were performed using SPSS version 19.0 (IBM Corporation, Somers, NY, USA). Comparisons between the two groups were made by using Fisher’s exact test. The relation between the age of patients and evaluated parameters was assessed using Pearson’s coefficient analysis. The number of participants was calculated on the basis of the effects observed in the previous study of IBS patients to achieve 80% power, in order to demonstrate significance with a *p*-value < 0.05 [35].

## 3. Results

### 3.1. Patients

The study included 244 patients with recognized IBS-D, who were predominantly women. The patients’ characteristics are presented in Table 1. The statistically significant differences between women and men concerned growth, body weight, and BMI. There were no statistical differences in terms of age and IBS severity between the women and the men. The patients who qualified for the study did not have any comorbidities according to the inclusion criteria, described in the Section 2.1. 

### 3.2. The Occurrence of Antibodies in IBS-D Patients

The results of the serological examination of the antibodies in the IBS-D patients are presented in Table 2. Elevated antibody concentrations, irrespective of the class of tested antibody, occurred in 44 patients (17.6%), including 31 women (17.7%) and 13 men (17.6%). Of these patients more than 1/3 (18, 40.9% of the patients with positive results for antibodies; 7.4% of the total number of patients) had elevated concentrations of antibodies associated with gluten consumption, which may indicate CD, allergy, or NCGS. The Pearson’s analysis did not show statistically significant correlations between age and the occurrence and concentrations of antibodies or IBS severity (data not presented).

#### 3.2.1. TTG2 and DGP Antibodies

Positive results for TTG2 IgA were detected in four IBS-D patients (1.64%) (Table 2), and three of these patients were positive for DGP IgA and IgG. There were two subjects with high TTG2 IgA concentrations (>100 kU/l): one woman aged 29.4 years and one man aged 34.0 years. We found three women (1.2% of the total patients, 1.8% of the total number of women) with IgA deficiencies; however, none of them were positive for TTG2 IgG, although one was found to be positive for DGP IgG (1.5 kU/L). Small intestinal specimens were obtained from three patients (all with positive TTG2 IgA antibodies), and the histological examinations showed typical CD morphological changes—increased numbers of intraepithelial lymphocytes (>25/100 enterocytes) and villous atrophy. The patient with the IgA deficiency and positive DGP IgG antibodies was not referred for endoscopy. One woman positive for TTG2 IgA refused endoscopy, but started self-administrated GFD, which improved the clinical manifestation of the disease. 

Additionally, 40 patients (16.4%) showed equivocal results (0.3–0.8 kU/L) for TTG2 IgA (*n* = 17) and TTG2 IgG (*n* = 23). Six of them (15%) were positive for DGP IgG antibodies.

Positive DGP antibodies (regardless of the class) were found in 11 patients (4.5% of total), without statistical differences between the women and the men. In most of the cases, DGP IgG antibodies were detected, and only three of these patients had elevated levels of anti-DGP antibodies in both classes.

Equivocal concentrations of DGP antibodies (regardless of the class) were found in 19 patients (7.8% of total).

#### 3.2.2. The sIgE Antibodies to Selected Food Allergens

We found sIgE (regardless of allergen) in 31 IBS-D patients (12.7% of the total; 11.8% of the women vs. 14.9% of the men) (Table 2). The highest percentage of positive sIgE was recorded in relation to the BSA (9.0%). Ten of the sIgE-positive patients (34.48%) had positive reactions to both the extract of cow’s milk and the BSA. Although the women (*n* = 8) were predominant in this group, the highest concentrations of sIgE were found in two men (sIgE to proteins from cow’s milk, 36 kU/L and 16 kU/L, and to BSA, 250 kU/L and 83 kU/L, respectively). Of the patients with sIgE to the extract from cow’s milk, only one man had sIgE to a specific component of milk, i.e., to alpha-lactalbumin (0.5 kU/L). No positive sIgE to beta-lactoglobulin or casein was detected. One woman and two men showed sIgE to the extract from cow’s milk only.

We detected sIgE to gluten in 2.05% of the patients, and this was the only result in which there was a statistically significant difference (*p* = 0.03) in favor of the men. In this group, none of the patients had positive CD-specific TTG2 IgA. 

#### 3.2.3. IF Antibodies

Positive IF IgG was found in six IBS-D patients (2.5% of the total; 2.9% of the women vs. 1.4% of the men). Among all the IF-IgG-positive patients, only one woman had positive DGP IgG antibodies. 

Additionally, 66 IBS-D patients (27.05% of the total, 30.59% of the women vs. 18.92% of the men) showed equivocal results (0.3–0.8 kU/L) for IF IgG. Of these, only one man was positive for TTG2 (IgA and IgG) and DGP (IgA and IgG) antibodies. Moreover, four women were positive for DGP IgG antibodies.

## 4. Discussion

Due to their clinical symptoms, adult patients qualifying as suffering from IBS-D may overlap with patients with CD, NCGS, food allergies, and AIG [7,8,9,33]. Our comprehensive analysis of the occurrence of serum TTG2 autoantibodies, DGP antibodies, IF autoantibodies, and sIgE to selected food allergens (gluten, proteins from cow’s milk, and BSA) showed a high percentage (17,6%) of IBS-D patients with positive results. Four of these participants had increased levels of TTG-2 IgA (1.6%), and in all of them, CD was recognized. Other authors found that the prevalence of CD in IBS patients ranged from 2.5% to 6.5% [40,41,42], and a meta-analysis of studies screening for CD in IBS demonstrated that the prevalence of biopsy-proven CD was 4.48% [7]. Thus, our results showed a lower percentage of recognized CD in IBS-D patients, although it is worth noting that the occurrence of CD was one of the exclusion criteria from the clinical trials in which the IBS-D patients were involved. Nevertheless, we were able to find patients with undiagnosed CD. Considering the fact that the pooled global prevalence of CD in the European population is about 0.8% [43], our results indicate the need for serological screening for CD in IBS patients, and are in line with the ACG clinical guidelines [40] and Polish recommendations for IBS patients [44]. Interestingly, we found that 16.4% of the IBS-D patients had TTG2 IgA in an equivocal range. These results do not authorize the patient to be referred for endoscopy in order to confirm the diagnosis of CD. However, it is necessary to remember that the performance of a single serological test for CD is insufficient, and CD screening tests should be repeated, especially in at-risk groups [45]. Thus, it cannot be ruled out that in patients with equivocal values of CD-specific antibodies, a further follow-up through serological CD screening would allow the recognition of new CD.

In our group of IBS-D patients, we found that 4.5% had positive DGP antibodies, but did not have CD-specific TTG2 autoantibodies. According to the recent CD diagnostic guidelines, such patients with normal total IgA levels should not be referred for endoscopy in order to obtain biopsies for histological examination [22,26]. However, the clinical significance of this phenomenon is unknown. We can only speculate that some of these patients could have NCGS. The general prevalence of NCGS in the population ranges between 0.6% and 10.6% [8,32]. This huge variability is mainly explained by the lack of diagnostic biomarker(s) and diagnosis based on the initial exclusion of CD and allergy to gluten, and the improvement of symptoms after the introduction of GFD. The research data that can be compared to our results are limited, because the presence of AGA is most often tested in IBS patients [7,46]. Lu et al. determined DGP IgA antibodies in an Asian population and found them in 18% of IBS patients [33]. Domżał-Magrowska et al., in studies performed on a small group of 48 IBS patients, found positive DGP IgA and IgG in 8.33% and 6.25% of these IBS patients, respectively [47]. However, in both studies, no GFD was introduced in the positive patients to show the clinical effect of this dietary challenge.

It is worth noting that in our studies, we found a relatively high percentage of patients not only with equivocal TTG2 IgA (as mentioned above), but also with equivocal TTG2 IgG and DGP IgG. These results indicate the presence of antibodies in the patients, but at concentrations that were not relevant to the patients’ clinical condition. However, the question arises as to whether limiting gluten in the diets of these patients would have an impact on their clinical condition. Currently, the low-FODMAP diet is recommended for IBS, but the effectiveness of this treatment is limited [40,48,49]. It needs to be highlighted that in low-FODMAP diets, cereals (including wheat) are often limited, but it is not known whether the beneficial effects of this diet can be observed in patients with positive gluten-related antibodies or in other IBS subgroups.

Our analysis also showed a high proportion (12.7%) of IBS-D patients sensitized to selected food allergens: gluten, proteins form cow’s milk, and BSA. Interestingly, more than 2% of the patients had sIgE to gluten. Gluten contains cereal prolamins, such as omega-5-gliadin, which is one of the most significant allergens causing gut symptoms that may overlap with IBS-D [50]. This protein is soluble in alcohol, which means that it may not be present in standard extracts for detecting sIgE to wheat flour. The immunoassay used in our study measures sIgE to gluten (as well as to omega-5-gliadin), not to whole extracts of wheat. Thus, the ability to determine sIgE to gluten, especially to omega-5-gliadin, significantly increases the diagnostic sensitivity of wheat allergies.

The highest percentage of positivity (9.0%) was recorded for the sIgE to BSA. Unfortunately, the available research does not provide information on the prevalence of sIgE to BSA in IBS patients. A fraction of whey from cow’s milk, BSA belongs to the group of serum albumins found in milk, serum, dander, and meat from mammals. Furthermore, BSA is a heat-sensitive allergen, which means that patients allergic to this protein may have gastrointestinal symptoms, such as abdominal pain, bloating, or diarrhea after drinking raw milk or eating uncooked meat [50]. Therefore, based on our results, it seems that it is worth conducting an in-depth analysis of the occurrence of allergy to proteins from cow’s milk in a group of patients with IBS-D.

The multiparametric immunoassay used in the current study allowed the detection of IF IgG, and these autoantibodies were found in 2.5% of the IBS-D patients. As there are no data on the occurrence of IF IgG autoantibodies in IBD patients, further research is needed to understand the clinical aspects of this finding. However, these antibodies are associated with AIG [34,51]. Due to the similarity between clinical symptoms such as abdominal pain and chronic diarrhea in AIG and IBS, it seems that the assessment of IF antibodies related to AIG may be justified in IBS patients.

In summary, the results showed a high percentage of IBS-D patients with positive tests assessing antibodies related to gluten, other food proteins, and anti-IF autoantibodies. Thus, serological screening for CD and food allergies seems to be a very important procedure for the differential diagnosis of IBS. In this context, it seems important to perform serological testing before the inclusion of IBS-D patients in clinical trials, especially those associated with dietary interventions, e.g., probiotic supplementation.

### Strengths and Limitations of the Study

The strength of this study is the comprehensive assessment of the presence of various antibodies associated with diseases (CD, NCGS, food allergy, and AIG) whose symptoms may be similar to those of IBS-D. The limitations of the study include the small size of the study group and the lack of a control group. The study mainly focused on the diagnosis of CD, but only patients with CD-specific positive TTG2 IgA were referred for small-intestinal biopsy and histological examinations. These examinations were not performed on the patients with positive DGP antibodies, especially one patient with IgA deficiency who was positive for DGP IgG, but negative for TTG2 IgG. There were also no under-diagnoses of other diseases or clinical observations of patients diagnosed with CD. Moreover due to the patient’s refusal to undergo an endoscopic examination and a biopsy of the intestine, it was not possible to perform a correlation of the serological with the histological finding. The specific limitation of the current study is the differences between the IBS diagnostic criteria (the Rome III and Rome IV criteria). Moreover, the use of a limited number of food allergens in the test restricted the analysis.

## 5. Conclusions

The current study showed a high prevalence of TTG2 autoantibodies, DGP antibodies, sIgE to selected food allergens, such as gluten, proteins from cow’s milk, and BSA, and IF autoantibodies in the group of IBS-D patients. Since the symptoms of IBS-D, CD, food allergies, and AIG can overlap, it is important to perform a proper differential diagnosis of IBS, and serological tests could be helpful in this process. However, further studies are needed to confirm the results obtained.

## Figures and Tables

**Table 1:** Patients’ characteristics.

	Women N (%) or Mean ± SD	Men N (%) or Mean ± SD
Number of patients	170 (69.7%)	74 (30.3%)
Age (years)	41.2 ±14.8	36.2 ±13.2
Growth (cm)	165.0 ± 5.7	177.7 ± 7.1 *
Body weight (kg)	67.8 ±13.6	85.2 ± 8.6 *
BMI	24.1 ±8.3	26.7 ±8.8 *
IBS severity		
Moderate	68 (40,0%)	32 (43,2%)
Severe	102 (60,0%)	42 (56,8%)
Total IBS-SSS score	339.8 ± 65.1	325 ± 76.8

* *p*-value < 0.001; SD = standard deviation; BMI = body-mass index; IBS severity was based on IBS-SSS scale. Severe IBS was identified when IBS-SSS score was >300, and moderate was identified when IBS-SSS score was >175 and ≤300.

**Table 2:** The results of the serological examination of the antibodies in IBS-D patients.

Antibodies	All Patients *n* = 244	Women *n* = 170	Men *n* = 74
**Gluten-related antibodies**			
TTG2 IgA	4 (1.6%)	3 (1.8%)	1 (1.4%)
TTG2 IgG	3 (1.2%) *	1 (0.6%)	2 (2.7%)
DGP IgA	5 (2.1%)	3 (1.8%)	2 (2.7%)
DGP IgG	9 (3.7%)	8 (4.7%)	1 (1.4%)
**AIG-related antibodies**			
IF IgG	6 (2.5%)	5 (2.9%)	1 (1.4%)
**sIgE antibodies to selected food allergens**			
Food allergens:			
gluten	5 (2.1%)	1 (0.6%)	4 (5.4%) **
extract of cow’s milk	13 (5.3%)	9 (5.3%)	4 (5.4%)
casein	0	0	0
alpha-lactalbumin	1 (0.4%)	0	1 (1.4%)
beta-lactoglobulin	0	0	0
bovine serum albumin	22 (9.0%)	17 (10.0%) #	5 (6.8%)
The total number of IBS-D patients positive for tested antibodies	44 (17.6%)	31 (17.7%)	13 (17.6%)

* a positive result for TTG2 IgG were found in 1 man, who was also positive for TTG2 IgA; ** *p*-value = 0.03; # two of the participants had positive results for TTG2 IgA and DGP IgA/IgG antibodies.

## Data Availability

Data are unavailable due to privacy or ethical restrictions.
